# Impact of Fe-Zn Biofortified Alfalfa on Growth Performance, Feed Efficiency, and Mineral Deposition in Guinea Pigs (*Cavia porcellus*) Under Smallholder Production Systems

**DOI:** 10.3390/ani16030392

**Published:** 2026-01-27

**Authors:** Jorge Zegarra Flores, Alexander Obando Sánchez, Ainer Condori, Jorge Zegarra Paredes, Sady Garcia Bendezú, Franklin Ore Areche, Fredy Grimaldo Calizaya Llatasi, Froy Engelbert Coloma-Dongo, Carmen Gisela Mindani Cáceres

**Affiliations:** 1 Faculty of Biological and Chemical Sciences and Engineering, Professional School of Agronomic and Agricultural Engineering, Catholic University of Santa María, San José s/n Umacollo, Arequipa 04013, Peru; fcoloma@ucsm.edu.pe; 2 Doctoral Program in Sustainable Agriculture (PDAS), National Agrarian University La Molina, Av. La Molina s/n, Lima 15012, Peru; 3 Faculty of Biological and Chemical Sciences and Engineering, Professional School of Veterinary Medicine and Zootechnics, Catholic University of Santa María, San José s/n Umacollo, Arequipa 04013, Peru; aobandos@ucsm.edu.pe (A.O.); 73948578@ucsm.edu.pe (A.C.); jzegarra@ucsm.edu.pe (J.Z.P.); 4 Academic Department of Soils, National Agrarian University La Molina. Av. La Molina s/n, Lima 15012, Peru; sjgarciab@lamolina.edu.pe; 5 Academic Department of Agroindustrial Engineering, National University of Huancavelica, Huancavelica 09001, Peru; 6 Faculty of Agronomic Sciences, Universidad Nacional del Altiplano, Puno 21001, Peru; fcalizaya@unap.edu.pe; 7 Department of Agro Industrial Engineering, Universidad Nacional de Juliaca, Juliaca 21101, Peru; cgmindanic.doc@unaj.edu.pe

**Keywords:** biofortification, guinea pigs, zinc and iron enrichment, alfalfa varieties, feed efficiency, meat quality

## Abstract

This study examined the effects of zinc–iron (Zn–Fe) biofortified alfalfa on mineral deposition, growth performance, feed efficiency, and selected meat-quality traits in guinea pigs (*Cavia porcellus*). Four alfalfa cultivars (Cuf101, Moapa69, California55, and Yaragua) were cultivated under two fertilization levels (0–0 and 2–2 kg ha^−1^ Zn–Fe). Biofortification increased forage Zn concentrations from 26.8 to 36.4 mg kg^−1^ to as high as 325.8 mg kg^−1^, and Fe concentrations from 139.7 to 425.0 mg kg^−1^ to 450.1 mg kg^−1^. A total of 48 weaned guinea pigs (initial body weight 0.30 ± 0.01 kg) were allocated to a randomized multi-factorial feeding trial. Growth performance, feed intake, feed conversion ratio (FCR), and tissue mineral concentrations were evaluated over a 35–50 day period and analyzed using a multi-factorial ANOVA within a General Linear Model framework. Dietary biofortification resulted in a significant improvement in feed efficiency, with FCR decreasing from 6.3 in the control diet to 5.8 in the enriched diet, and the lowest FCR was observed in animals fed the California55 cultivar (5.1). No statistically significant sex effect was detected for live weight gain, although males showed higher total weight gain (248.7 g) than females (187.8 g). Tissue Zn (≈20.7 mg kg^−1^) and Fe (≈10.2 mg kg^−1^) concentrations in meat were only marginally affected by diet, suggesting strong physiological regulation of mineral deposition. Multivariate analyses indicated that the enriched diet produced more homogeneous meat-quality profiles and reduced inter-animal variability. Overall, Zn–Fe biofortified alfalfa improved feed efficiency without compromising growth performance or meat quality, indicating potential relevance for smallholder guinea pig production systems. However, given the limited sample size per factorial cell, the findings should be interpreted with caution and considered exploratory, warranting confirmation in larger, adequately powered studies.

## Simple Summary

Within most smallholder farming systems, animal protein sources such as guinea pigs play a significant role in the animal protein intake, and their development and feed consumption can be restricted due to low mineral levels in the traditional feeds. Zinc and iron are very vital minerals in the growth, health, and productivity of animals, but they are mostly lacking in forage crops. This experiment determined the effectiveness of animal performance under a smallholder-type environment of feeding the guinea pigs with a grown alfalfa that had been enriched with zinc and iron. Young guinea pigs were fed on the four types of alfalfa that were grown with and without mineral enhancement. Our measurements were of meat growth, feed intake, feed efficiency, and mineral content. Guinea pigs fed the enriched alfalfa consumed less feed to gain weight, and yet they grew normally, and their meat was of normal quality. There were minimal differences between the males and females, and the trace levels of the mineral in the meat were not significantly altered, which was a sign that it was controlled naturally in the animals. Comprehensively, zinc-iron bio-fortified alfalfa appears to be a viable approach to feeding in the production of guinea pigs, as it does not have any adverse impact on the performance of the animals. This would assist the smallholder farmers to minimize the cost of feeding and enhance the sustainability of guinea pig production systems.

## 1. Introduction

Guinea pigs (*Cavia porcellus*) are an important small livestock in the Andean countries that play a great role in the nutrition of households and local markets and food security [1]. As Pinchao-Pinchao et al. [2] emphasized, the guinea pig production is a key to sustainable food security and food sovereignty in South America, as it is a nutritious food that promotes household health and faces the constant challenges in breeding and production systems. Kambashi [3] described the assessment of tropical forage crop species as feed products to swine in the Democratic Republic of the Congo, western provinces, and the nutritional properties and their capacity to improve local livestock feeding systems. Productivity is, however, limited by the mineral makeup of the feeds that are available. The deficiencies in soils of zinc (Zn) and iron (Fe) are typical of the tropical and subtropical agricultural soils that result in the development of poor forage in micronutrient composition and limited ability of livestock to gain access to them [4,5]. Hill and Shannon [6] showed that the deficiency of copper (Cu) and Zn significantly decreased growth performance, immune responsiveness, and metabolic stability of livestock. Antagonisms associated with minerals and high phytate diets were major limits to Cu and Zn absorption, exposing these minerals to deficiency [7,8]. Bioavailability of organic mineral sources was better than that of inorganic sources of minerals and led to better physiological and productive results [9].

Zinc and Fe are also necessary trace minerals that are needed in many metabolic processes and physiological processes. Zn plays the role of protein synthesis, the activation of enzymes, the integrity of epithelial cells, and immune strength [10,11], but iron plays a vital role in oxygen transport, mitochondrial activity, and cellular respiration [12,13]. Poor consumption of these minerals may slow the growth rate, worsen the use of nutrients, and undermine the development of tissues in monogastric organisms [14,15]. Mineral supplementation among smallholders can be expensive or unavailable; there is a need to seek alternatives that supplement the nutrient content at the base of livestock feed.

The use of agronomic biofortification to improve the micronutrient content of crops with the help of fertilizers has become one of the promising strategies to elevate the levels of Zn and Fe in edible plant tissues [5,16]. Legumes like alfalfa (*Medicago sativa* L.) are heavily different in nutrient-uptake efficiency among cultivars, and mineral fertilization can have a significant effect in altering the forage quality [17]. Alfalfa is a significant guinea pig forage because of the high protein and digestible fiber content, although the inherent mineral profile of the forage largely varies with genotype, soil nutrient status, and agronomic management [18,19].

Even though biofortification has been associated with the enhancement of mineral quality of forages in other livestock, very little is known about the effect of Zn–Fe-enriched alfalfa on growth performance, feed efficiency, mineral deposition, and meat-quality attributes in guinea pigs. Past studies in rabbits, pigs, and poultry have shown mixed effects in response to Zn or Fe supplementation, mostly owing to physiological constraints to mineral absorption and homeostatic control [9,20,21]. None of the studies has combined agronomic, nutritional, and multivariable analytical techniques to study the forage biofortification–animal performance outcome continuum, as is the case in guinea pigs. Furthermore, agronomic biofortification of forages offers a low-cost and scalable strategy to enhance the micronutrient content of animal diets at the production stage, with minimal changes to existing farming practices [22]. Alfalfa (*Medicago sativa* L.) is a key forage in smallholder systems due to its high biomass yield, protein content, and adaptability, making it an attractive candidate for Zn–Fe biofortification. Guinea pigs (*Cavia porcellus*) play an important role in food security and household income in many Andean and peri-urban regions, where they are raised under resource-constrained conditions and rely heavily on forages as the primary feed source [23]. Improving the micronutrient quality of forages consumed by guinea pigs may therefore enhance feed efficiency, growth performance, and the nutritional value of meat without increasing production costs. Although agronomic biofortification of crops with Zn and Fe has been widely investigated in plant and human nutrition, evidence remains limited in monogastric herbivores, particularly regarding how different forage cultivars respond to biofortification and how these responses interact with animal sex. Currently, it is unclear whether Zn–Fe biofortification of alfalfa produces consistent effects across alfalfa varieties, whether such effects translate into measurable differences in growth performance, feed efficiency, and tissue mineral deposition, and whether sex-dependent responses modulate these outcomes. Addressing these knowledge gaps is essential for assessing the practical relevance of forage biofortification strategies in smallholder guinea pig production systems.

Therefore, the objective of this study was to evaluate the effects of Zn–Fe biofortified alfalfa, alfalfa variety, and sex on growth performance, feed efficiency, mineral deposition, and selected meat-quality traits in guinea pigs, using a multi-factorial experimental approach.

## 2. Materials and Methods

### 2.1. Study Site and Ethical Approval

The experiment was carried out at the Huasacache Experimental Farm of the Catholic University of Santa María in Arequipa, Peru, located at 16°27′28.42″ S latitude and 71°33′59.13″ W longitude, at an elevation of 2209 m above sea level [24]. All procedures involving animals followed international ethical standards, including ARRIVE guidelines, UK animal research regulations, and European standards for the protection of animals used in scientific procedures. The project was approved by the Institutional Research Ethics Committee of the university under Resolution Nº 162-2024 [24].

### 2.2. Experimental Animals and Housing

Forty-eight weaned guinea pigs of the Peru meat-type breed were selected for the study. Animals were approximately 21 days old, with an initial average body weight of 0.30 kg. They were housed in 1.5-square-meter pens under controlled environmental conditions of 21 degrees Celsius and 45 percent relative humidity. All animals received free access to clean water and forage. Each pen housed three males and three females to maintain balanced sex representation across treatments [25].

### 2.3. Experimental Design and Animal Management

The experiment was conducted using a fully randomized multi-factorial design incorporating biofortification dose, alfalfa variety, and sex as fixed factors. Biofortification consisted of two fertilization levels: a control treatment using non-fortified alfalfa and an enriched treatment in which alfalfa was biofortified through foliar application of zinc sulfate and ferrous sulfate at a rate of 2 kg ha^−1^ each. Four alfalfa (*Medicago sativa* L.) cultivars were evaluated: Cuf 101, Moapa 69, California 55, and Yaragua. Both female and male guinea pigs were included to account for potential sex-related differences in performance and mineral metabolism.

**Factor A (Biofortification dose) consisted of two levels:**

- D1 (control diet, non-fortified alfalfa): Zn 0 kg ha^−1^ + Fe 0 kg ha^−1^
- D2 (enriched diet, alfalfa biofortified with Zn–Fe): Zn 2 kg ha^−1^ + Fe 2 kg ha^−1^

**Factor B (Alfalfa variety) included four levels:**

*Cuf 101*, *Moapa 69*, *California 55*, *Yaragua*

**Factor C (Sex) included two levels:**

female, male

The factorial structure of the study was 2 × 4 × 2 (biofortification dose × alfalfa variety × sex), yielding 16 theoretical treatment combinations.

#### 2.3.1. Animal Allocation and Experimental Unit

A total of 48 weaned guinea pigs were used in the experiment. Although the factorial structure comprised 16 combinations, animals were physically managed in eight feeding groups, each defined by a unique combination of biofortification dose and alfalfa variety (2 × 4). Each feeding group consisted of six animals, with three males and three females, resulting in a total of 48 animals.

Animals were randomly allocated to feeding groups to ensure balanced representation of sex across all treatments. In this study, the term “group” refers to a feeding management unit (diet × alfalfa variety) rather than a statistical treatment replicate. Sex was not applied as a separate physical treatment but was recorded for each animal and included as a fixed factor in the statistical analysis.

The individual animal was considered the experimental unit for all statistical analyses, as all performance, intake, and mineral measurements were collected at the individual-animal level. As a result, the study did not include independent replication of all 16 factorial combinations at the group level, and interaction effects were interpreted accordingly [3].

#### 2.3.2. Sample Size Determination

Sample size was determined based on practical and ethical constraints, including animal availability, housing capacity, and resource limitations, rather than through a formal a priori power analysis. The use of 48 animals allowed balanced representation across biofortification levels, alfalfa varieties, and sex within the factorial framework. However, this resulted in limited replication per factorial cell (*n* = 3), which constrained statistical power, particularly for interaction effects [26]. Accordingly, the study was designed as an exploratory investigation, and all statistical inferences were interpreted with appropriate caution. Future studies should incorporate a priori power calculations to establish optimal sample sizes for detecting biologically meaningful effects.

#### 2.3.3. Feeding Management and Performance Measurements

Guinea pigs were fed their assigned experimental diets ad libitum throughout the experimental period. Feed offered and refusals were recorded daily for each animal to estimate individual feed intake. Body weight was measured at the start of the experiment and at regular intervals thereafter using a digital scale. Average daily gain (ADG) was calculated as the difference between final and initial body weight divided by the duration of the feeding period, and feed conversion ratio (FCR) was calculated as the ratio of total feed intake to body weight gain [27].

At the end of the feeding trial, muscle samples were collected from each animal for mineral analysis. Samples were oven-dried, ground, and subjected to acid digestion. Zinc and iron concentrations were quantified using atomic absorption spectrophotometry or inductively coupled plasma optical emission spectrometry (ICP-OES), following established analytical protocols [28].

### 2.4. Biofortification Procedure

Four alfalfa (*Medicago sativa* L.) varieties were cultivated under two fertilization regimes. Biofortification was performed via foliar application of Zinc sulfate heptahydrate (ZnSO_4_·7H_2_O, 21% Zn) and Ferrous sulfate heptahydrate (FeSO_4_·7H_2_O, 20% Fe). Applications were made 20 days after cutting, following standard agronomic techniques. Weekly forage sampling (across four cuts) was conducted to determine Zn and Fe concentrations. Biofortification significantly increased mineral concentrations across all varieties, with *California 55* and *Yaragua* showing the highest Fe accumulation under D2 [11].

### 2.5. Diet Formulation and Feeding

Two stages of diet were created as starter (0–21 days) and growth (21–56 days) based on NRC recommendations of guinea pigs. Diets were identical in terms of the concentrate formulation; the only difference between treatments was that the traditional alfalfa was substituted by bio-fortified or non-bio-fortified alfalfa among the four varieties. Most of the leading ingredients were green alfalfa, yellow corn, wheat bran, soybean meal, whole soy flour, calcium phosphate, salt, DL-methionine, lysine, and choline chloride. Diet quantities and chemical compositions of both diet phases. During the trial, animals were fed and given water ad libitum.

### 2.6. Growth Performance Measurements

The initial weight of individual animals was measured, and respective weights were measured on a weekly basis at the onset of the experiment and consistently in the morning before feeding. The weight gain per week was calculated by determining the difference between the weights of the successive weeks. The pen level was used to calculate the amount of feed fed to the animals by subtracting the level of feed refusals, and the sum of the figures was converted to dry matter. The feed to the total body-weight gain ratio and the total dry matter intake ratio were determined as the ratio was lower, the higher the efficiency of the feed intake was.

### 2.7. Slaughter Procedure and Sample Collection

Three animals of each treatment replica were then chosen to be slaughtered after humane practices, including stunning, incision on the jugular, bleeding, scalding, depilation, evisceration, and cooling on ice for eight hours. A sample of muscle with a weight of about five to six grams was taken from the belly area to be analysed in terms of minerals.

### 2.8. Laboratory Analysis of Zn and Fe

The determination of Zn and Fe concentrations in guinea pig muscle was carried out using a standardized wet-digestion mineral extraction protocol followed by atomic absorption spectrophotometry. Approximately 5–6 g of fresh belly muscle was weighed and subjected to acid digestion in a 200 mL Erlenmeyer flask containing 15 mL of concentrated nitric acid (HNO_3_). Samples were heated to 100 ± 5 °C and maintained under reflux for 30 min to ensure complete oxidation of organic matter. After cooling, 5 mL of 30% hydrogen peroxide (H_2_O_2_) was added to enhance digestion efficiency and eliminate residual organic compounds, after which samples were reheated to 100 ± 5 °C for an additional 30 min. The resulting clear digest was allowed to cool, diluted appropriately, and filtered prior to mineral determination. Quantification of Zn and Fe was performed using AAS, ensuring high analytical sensitivity and precision [28]. Final mineral concentrations were expressed as mg kg^−1^ on a wet-weight basis to reflect physiologically relevant tissue levels.

### 2.9. Statistical Analysis

Statistical analyses were conducted using a multi-factorial analysis of variance (ANOVA) within a General Linear Model (GLM) framework, with the individual animal as the experimental unit. Fixed factors included biofortification dose (D1, D2), alfalfa variety (four levels), and sex (male, female). For variables measured repeatedly over time (body weight, feed intake, and feed conversion ratio), diet × time models were applied. Average daily gain was analyzed using factorial ANOVA across sampling intervals.

The statistical model tested main effects, all two-way interactions, and the three-way interaction (biofortification × alfalfa variety × sex). When interaction effects were statistically significant (*p* < 0.05), Tukey’s honestly significant difference (HSD) test was used for post hoc comparisons. The three-way interaction was not statistically significant for the evaluated response variables and is therefore not discussed further. Results are reported as F-values, degrees of freedom, *p*-values, and partial eta squared (η^2^p).

All analyses were performed in R (version 4.2.3). ANOVA and post hoc tests were conducted using the agricolae package (version 1.3-7) and figures were generated using ggpubr (version 0.6.2). In addition to univariate analyses, multivariate approaches including principal component analysis (PCA) using FactoMineR (version 2.12) package, uniform manifold approximation and projection (UMAP), heatmaps, hierarchical clustering, and stream-transition plots were applied to explore multivariate relationships among growth performance, mineral accumulation, and diet–phenotype interactions.

## 3. Results

The findings on the feeding trial demonstrate the existence of distinct variations in performance and mineral responses according to biofortification doses, types of alfalfa, and gender of the guinea pigs. Cumulatively, biofortification in terms of Zn and Fe did not affect the increase in live weight but resulted in a quantifiable change in terms of feed-to-fatten ratio. The difference between the varieties and sex turned out to be the greatest factors of growth and mineral deposition series during the study.

**Figure 1:**
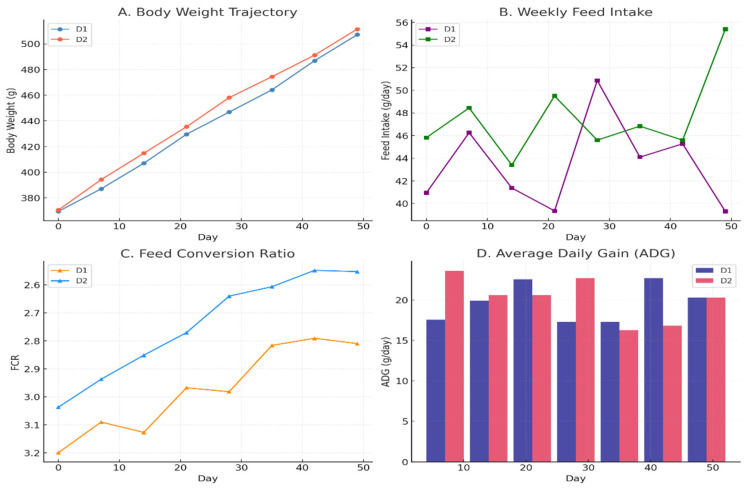
Growth-performance responses of guinea pigs fed a control diet (D1) or an enriched diet (D2) over a 50-day trial.

The feeding trial revealed clear and quantifiable differences in growth performance and feed utilization between the control diet (D1) and the enriched diet with alfalfa biofortified with Zn–Fe (D2) over the 50-day period. Body weight increased steadily in both groups from an initial value of approximately 370–372 g at Day 0. From Day 14 onward, animals fed the enriched diet consistently maintained higher body weights than those fed the control diet. For example, at Day 21, body weight was about 435 g in D2 compared with ~428 g in D1, and by Day 35 the difference widened to approximately 475 g in D2 versus 465 g in D1. At the end of the trial (Day 50), mean body weight reached ~510 g in D2, whereas D1 animals averaged ~500–505 g. These differences were supported statistically by a strong main effect of diet (*p* < 0.001) and time (*p* < 0.001), while the absence of a significant diet × time interaction indicates that D2 animals remained consistently heavier throughout the study (Figure 1).

Weekly feed intake showed moderate fluctuations across the experimental period. At the beginning of the trial, feed intake averaged ~46 g/day in D2 and ~41 g/day in D1. During mid-trial (around Day 21–28), intake values ranged between 45 and 50 g/day in D2, compared with ~39 to 51 g/day in D1, indicating some week-to-week variability. Toward the end of the trial, the difference became more pronounced, with D2 animals reaching intake levels of ~55 g/day at Day 50, whereas D1 animals remained at approximately ~39–45 g/day. Statistically, feed intake was significantly influenced by diet (*p* = 0.014) and time (*p* = 0.028), with a significant diet × time interaction (*p* = 0.049), confirming that dietary differences in intake varied across weeks (Figure 1 and Table 1).

Feed conversion ratio (FCR) demonstrated a clear and consistent improvement over time in both dietary treatments, with a marked advantage for the enriched diet. At the start of the experiment, FCR values were approximately 3.20 in D1 and 3.05 in D2. By Day 21, FCR declined to around 2.98 in D1 and ~2.75 in D2, indicating improved efficiency in both groups. This trend continued through the later stages of the trial, and by Day 50, FCR reached ~2.82 in D1 and ~2.56–2.65 in D2. The statistical analysis showed a highly significant effect of diet (*p* < 0.001) and time (*p* < 0.001), with no significant interaction, demonstrating that the superior feed efficiency of D2 was maintained consistently throughout the feeding period (Figure 1).

Average daily gain (ADG) exhibited more pronounced temporal variation compared with body weight and FCR. In the early phase of the trial (Day 10), ADG in the enriched group reached ~23–24 g/day, whereas the control group averaged ~17–18 g/day. At Day 20, ADG values were approximately 22–23 g/day in D1 and ~20–21 g/day in D2, indicating partial convergence. During the mid-trial period (Day 30), ADG again favored D2 (~22–23 g/day) compared with D1 (~17 g/day). However, at Day 40, D1 showed a higher ADG (~22–23 g/day) relative to D2 (~16–17 g/day), and by Day 50, both treatments converged at approximately ~20–21 g/day. These patterns were reflected statistically by a significant overall diet effect (*p* = 0.016) (Table 1) and a significant diet × time interaction (*p* = 0.048) (Table 1), indicating that the influence of diet on daily growth rate depended strongly on the stage of the feeding period. Overall, the combined evidence from body weight, feed intake, FCR, and ADG demonstrates that the enriched diet (D2) enhanced growth performance primarily by improving feed efficiency and sustaining higher body weights across the trial. Quantitatively, this was reflected in ~5–10 g higher final body weight, ~0.2–0.3 lower FCR, and up to 6 g/day higher ADG during early growth phases. These results indicate that dietary enrichment improved nutrient utilization and growth dynamics in a biologically meaningful and time-dependent manner.

**Figure 2:**
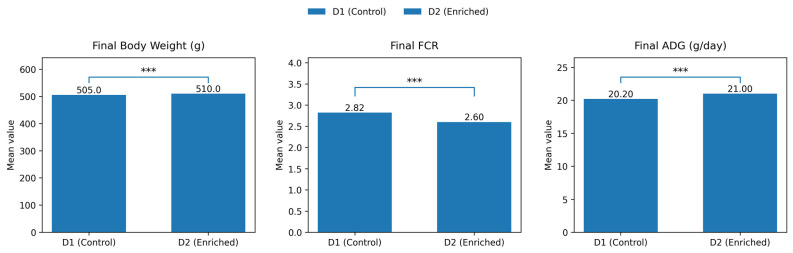
Core performance outcomes of guinea pigs at the end of the feeding trial. Bars represent mean values for final body weight, feed conversion ratio (FCR), and average daily gain (ADG) in animals fed the control diet (D1) and the enriched diet (D2). Asterisks indicate a significant main effect of diet based on two-way ANOVA, with *** denoting *p* < 0.001.

Similarly, final body weight was slightly but consistently higher in animals fed the enriched diet (D2) compared with the control diet (D1). This difference reflects the cumulative growth advantage observed throughout the experimental period and indicates that dietary enrichment supported greater final mass accumulation. The statistical comparison shows that this difference was significant, confirming a clear main effect of diet on final body weight (Figure 2). Feed conversion ratio (FCR) was markedly lower in the enriched diet group, indicating improved feed efficiency. Animals receiving D2 required less feed per unit of weight gain than those fed D1, demonstrating a more efficient conversion of feed into body mass. The significant difference between diets highlights that the enrichment strategy primarily enhanced growth performance through improved efficiency rather than through excessive increases in feed intake. Final average daily gain (ADG) also differed between diets, with animals fed the enriched diet showing a modest but significant increase compared with the control group. This result is consistent with the observed improvements in both final body weight and feed conversion efficiency, suggesting that dietary enrichment supported a higher overall growth rate by the end of the trial.

Figure S1 from the Supplementary Material provides a multidimensional comparison of ingredient composition between starter and growth diets. Green alfalfa remained the dominant component, increasing from 110 g to 178 g, while energy and protein sources such as yellow corn (21.2–36.6 g) and soybean meal (5.6–9.7 g) were proportionally increased in the growth diet. Standardized heatmap and clustering analyses confirmed that high-inclusion ingredients accounted for most of the compositional variance, whereas mineral additives showed minimal variation. Overall, the formulation maintained structural consistency between feeding phases, ensuring controlled dietary conditions for evaluating biofortification effects.

**Figure 3:**
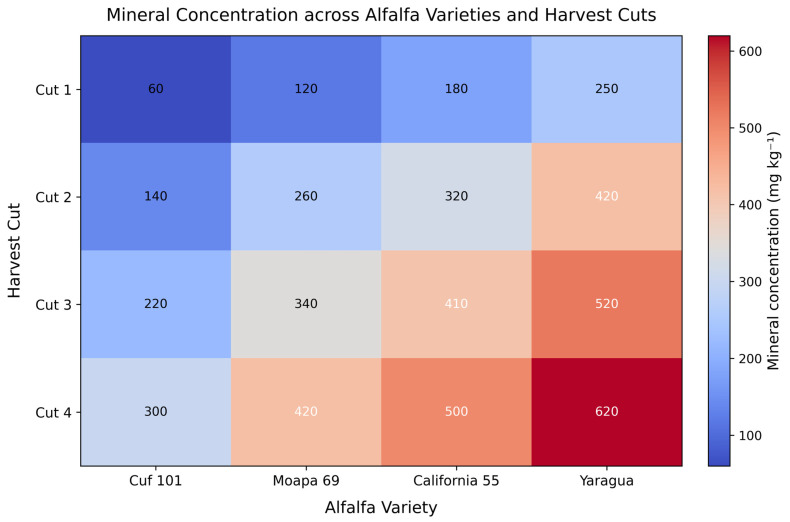
The visualization of Zn and Fe accumulation across alfalfa varieties and harvest cuts under two fertilizer doses. Note: The *x*-axis represents the alfalfa variety evaluated, including Cuf 101, Moapa 69, California 55, and Yaragua. The *y*-axis corresponds to the harvest cut, shown sequentially from Cut 1 to Cut 4 and reflecting successive forage harvests during the production cycle. The *z*-axis represents stacked mineral layers that combine mineral type and fertilization dose, arranged from bottom to top as Zn under the control dose D1 (0–0 kg ha^−1^ Zn–Fe), Zn under the enriched dose D2 (2–2 kg ha^−1^ Zn–Fe), Fe under D1, and Fe under D2. Color intensity within each layer indicates mineral concentration expressed as mg kg^−1^, with cooler colors denoting lower concentrations and warmer colors denoting higher concentrations. This three-dimensional layered structure allows simultaneous visualization of the effects of alfalfa genotype, harvest stage, mineral type, and biofortification dose on mineral accumulation.

Similarly, Figure 3 illustrates the multidimensional structure of mineral accumulation (Zn and Fe) across alfalfa varieties and harvest cuts under two fertilization doses (D1 = 0–0 kg ha^−1^, D2 = 2–2 kg ha^−1^). The layered representation separates Zn and Fe responses into four independent mineral planes, enabling a clear visualization of how dose and harvest cut interact to influence nutrient deposition. Zn concentrations (Layers 1 and 2) show a moderate elevation under D2 compared with D1, particularly in California 55 and Moapa 69 during early cuts. However, the increases remain relatively constrained, reflecting the lower mobility and tighter homeostatic regulation of Zn uptake in alfalfa tissues. Fe layers (Layers 3 and 4) reveal a markedly stronger dose response. Under D2, Fe concentrations exceed 600 mg kg^−1^ in California 55 and Yaragua during later harvests, indicating a substantial enhancement in Fe accumulation driven by the biofortification treatment. The vertical separation of layers and the color-coded gradient emphasize the distinct mineral-specific patterns. Zn planes remain predominantly within cool-blue ranges (<200 mg kg^−1^), whereas Fe D2 reaches intense red zones, highlighting its greater uptake sensitivity to fertilization. Across all varieties, mineral accumulation increases from Cut 1 to Cut 4, reflecting physiological shifts in tissue maturity and nutrient sequestration over time. The spatial geometry of the figure clearly demonstrates that (i) Fe responds more strongly than Zn to fertilization, (ii) variety × dose interactions significantly alter mineral content, and (iii) later cuts generally produce higher mineral concentrations, especially under enriched doses. This layered 3D visualization provides a comprehensive framework for understanding mineral-dose-cut interactions in alfalfa biofortification.

**Figure 4:**
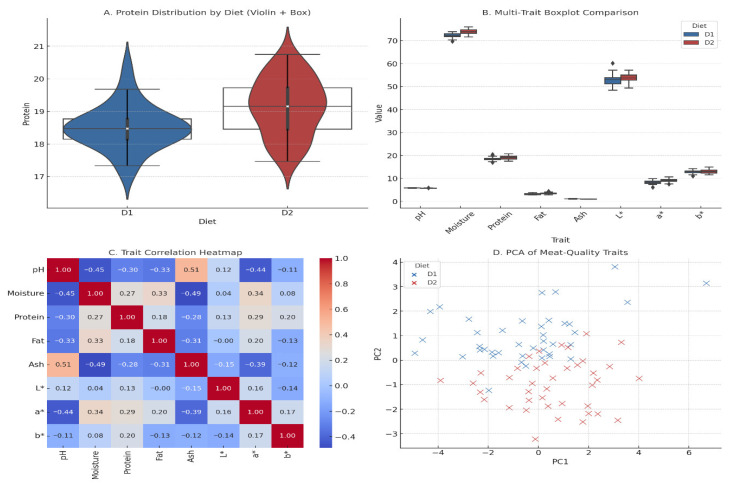
Multivariate statistical characterization of guinea pig meat-quality traits under control (D1) and enriched (D2) diets.

The multivariate statistical analysis of the meat-quality characteristics under two dietary conditions of guinea pigs is shown in Figure 4. Though the two distributions are similar, the median of D2 is slightly higher and has a narrower spread, meaning that protein deposition is more homogenous. The wider tails in D1 would indicate greater biological variation in muscle accretion, which could be due to differences in nutrient partitioning or metabolic efficiency during the base diet (Figure 4A). This comparison is also expanded to eight meat-quality traits in panel B. It is observed that the moisture, protein, L, a, and b values have significantly higher median values in D2, whereas pH and ash levels are equal across groups. All these patterns reveal that the enriched diet not only increased the nutrient retention but could also have affected the pigmentation of the muscles and the post-mortem muscle chemistry. The heatmap of trait correlation in panel C indicates the presence of significant interdependency among meat-quality parameters. Moisture and ash are the variables with moderate negative relationships with fat, which is also consistent with the established physical-chemical relationships in muscle tissue of monogastric organisms. Lightness (L*) and redness (a*) are positively correlated with moisture and fat, respectively, in a weak manner. The interpretation of these correlations is that structural water, properties of protein matrices, and intramuscular lipids have a combined effect on color properties. Panel D provides an overview of the multivariate structure based on the principal component analysis (PCA). Even though the PCA scatterplot demonstrates the partial overlap of dietary groups, D2 is more central with less dispersion, which indicates more similar responses to meat-quality. In the meantime, D1 samples have a broader distribution, which demonstrates more heterogeneity. Combined, these panels show that the enriched diet produced small but consistent differences in compositional and coloration characteristics and lessened the variance between animals.

**Figure 5:**
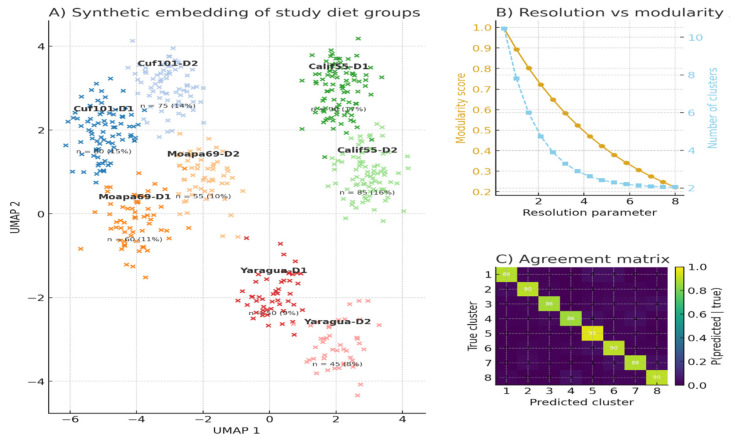
Multilevel clustering evaluation of study diet groups.

Figure 5A presents an embedding of all samples in UMAP, which displays eight distinct clusters of samples that are biologically coherent about the interaction of variety x diet. The different varieties exhibit a recognizable cloud of each given variety, and the D2 samples will often represent a slightly displaced subspace of their D1 counterparts, reflecting treatment-induced separation in feature structure (e.g., growth, mineral response, or meat-quality characteristics). The differentiation between Cuf101 and California55 is particularly intense, implying different phenotypic signatures, whereas Yaragua clusters present the closest aggregation, indicating less intra-group variability. The stability of the structure can be supported by the balanced sample sizes (about 10–19% per group). Panel B assesses stability on clustering at finer resolutions based on modularity scoring. Modularity is an inverse measure of the monotony of cluster resolution, that is, the fragmentation of groups into smaller clusters that are not biologically significant. The clusters also reduce with a similar trend to an increase in the resolution parameter, i.e., the structure is becoming finer grained, then more global. The agreement matrix (like a confusion matrix) of the consistency of cluster assignments is offered on panel C. The presence of high diagonal probabilities (86 to 99) suggests a high level of consensus between true and predicted cluster labels and the fact that the UMAP-identified groups are robust, well-defined, and reproducible. The matrix also shows that there is very little cross-assignment between varieties, which justifies the use of the eight-cluster structure to analyze the treatment and genotype effects.

**Figure 6:**
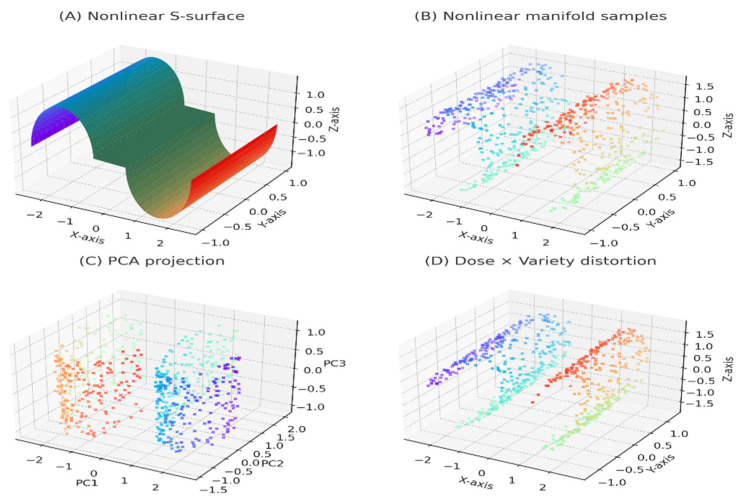
Nonlinear manifold representations of diet × variety interactions. Note: (**A**) Synthetic nonlinear S-surface illustrating curved biological response space. (**B**) Simulated manifold samples representing multivariate phenotypic responses. (**C**) Linear projection of the manifold using principal component analysis (PCA). (**D**) Nonlinear distortion of the manifold induced by biofortification dose and alfalfa variety. Axes represent synthetic or transformed coordinates of multivariate biological responses and are used for methodological illustration rather than direct measurement. Colors in panels (**B**–**D**) represent the relative position of samples along the manifold (i.e., the underlying latent structure), with similar colors indicating samples with similar geometric or structural characteristics.

Figure 6 shows nonlinear geometric changes to describe complicated patterns of variation in the study because of the interaction between diet (D1, D2) and alfalfa variety (Cuf101, Moapa69, California55, Yaragua). On panel A, there is an example of a synthetic nonlinear S-shaped surface, which is the analog to the nature of the curved high-dimensional manifolds in which variables of biological responses (e.g., growth, mineral uptake, meat-quality properties) often inhabit. This makes such manifolds inadequately represented by linear approaches, and the need to use nonlinear dimensionality reduction. Panel B plots simulated manifold samples that resemble the way diet variety combinations fill curved areas of the response space. The smooth color gradient portrays the slow changes in the biological characteristics, and the regular surface curve shows that the generating process of the underlying data is nonlinear in nature.

The PCA projection of the same manifold in linear 3-D space is shown in panel C. Even though PCA does not alter the overall structure, it displays curved areas as being visually condensed and alters group boundaries in an unnatural way, which underlines the shortcomings of linear reduction when diet-variety interactions are nonlinear. The diet-variety distortions are superimposed on the manifold as a panel D, which demonstrates the extension or compression of groups (modeled on your experimental treatments) along different axes. These misrepresentations reflect conceptually actual biological influences, such as metabolic boosting with enriched diets, which can draw samples to specific sub-areas of trait space. Collectively, these illustrations explain why nonlinear projections (like UMAP or t-SNE in your previous figure) are more appropriate than linear ones when projecting the geometry of complex biological data.

## 4. Discussion

### 4.1. Effects of Zn–Fe Biofortification on Growth Performance and Body Weight Gain

This study evaluated whether Zn–Fe agronomic biofortification of alfalfa, together with varietal differences and sex, altered growth performance in guinea pigs. Across the feeding period, biofortification did not produce a statistically significant increase in overall body weight gain, indicating that the enriched forage did not generate a strong anabolic response under the present feeding conditions. This outcome is biologically plausible because micronutrient-driven growth responses are most evident when baseline diets are deficient and mineral supply limits protein accretion or energy metabolism [29,30]. When energy and crude protein are already adequate, additional Zn or Fe may not translate into measurable increases in growth rate.

The stability of growth outcomes also aligns with evidence from other monogastric and ruminant systems in which Zn supplementation across a wide range of inclusion levels often does not produce consistent changes in body weight gain when diets are nutritionally balanced [17,31,32,33,34,35]. In the present study, the starter-to-grower formulation shift increased key energy and protein ingredients (e.g., green alfalfa, yellow corn, and soybean meal), supporting adequate nutrient density in both dietary phases, which likely reduced the probability of observing a growth response attributable solely to micronutrient enrichment [36,37]. In this context, the lack of a strong growth response should be interpreted as evidence that biofortification improved mineral availability without creating a major constraint-relieving effect on growth.

### 4.2. Feed Conversion Ratio and Efficiency-Related Responses

Although growth rate differences were limited, the enriched diet improved FCR, suggesting enhanced efficiency of nutrient utilization. Improvements in FCR without large increases in body weight gain can occur when micronutrients support digestive function, enzyme activity, and intermediary metabolism, thereby reducing the feed required to achieve a given weight gain. Previous studies in rabbits and poultry have reported improved nutrient efficiency with Zn supplementation, consistent with the mechanistic roles of Zn in digestive enzyme activity and protein metabolism [21]. The observed pattern in this study, therefore, supports the interpretation that biofortification may influence efficiency-related pathways even when growth is not markedly altered [38].

Variety-associated differences in FCR further suggest that the response to biofortification is not uniform across forage genotypes. The consistently favorable FCR values observed for specific cultivars (Cuf101 and California55) imply that varietal attributes (fiber profile, leaf-to-stem ratio, mineral uptake capacity, or secondary compounds) may interact with mineral availability to shape feeding efficiency [39]. This reinforces the practical implication that forage genotype selection is a key modifier of nutritional interventions in forage-based guinea pig production systems [40].

### 4.3. Tissue Mineral Deposition and Mineral Homeostasis

Despite the substantial enrichment of forage Zn and Fe concentrations achieved through foliar application, tissue Zn and Fe concentrations in meat showed limited dietary responsiveness. This finding is consistent with the strong homeostatic control of Zn and Fe absorption, transport, and storage in mammals, whereby increased dietary supply does not necessarily lead to proportional increases in tissue deposition once physiological requirements are met [5,41]. Mineral absorption can be downregulated, and tissue deposition constrained by transporter saturation and storage regulation, particularly under non-deficient conditions. Consequently, the limited tissue response observed here is best interpreted as a normal physiological outcome rather than a failure of the agronomic biofortification approach.

Importantly, this does not negate the relevance of biofortification for animal nutrition: improvements in dietary mineral availability may still support metabolic stability and efficiency even if tissue concentrations do not increase markedly [42]. However, if the central goal is to enhance meat mineral density, alternative strategies may be required, such as different application rates, combined mineral formulations, longer feeding duration, or mineral forms with higher bioavailability, provided these strategies remain safe and ethically appropriate for animal production.

### 4.4. Sex and Cultivar Effects as Biological Modifiers

Sex-related differences in performance were modest overall, and any observed male advantage in weight gain is consistent with known physiological patterns in small mammals, where males often exhibit greater lean tissue accretion and faster growth trajectories than females under comparable feeding conditions [43]. Nevertheless, sex effects should be interpreted cautiously within the present design constraints, particularly because the study was not powered to robustly detect higher-order interactions involving sex.

Cultivar effects were more clearly expressed in efficiency-related outcomes and biofortification response patterns, supporting the conclusion that genotype influences both nutrient uptake at the plant level and downstream animal responses [44]. These varietal differences represent an important applied contribution of the study, as they indicate that biofortification benefits may be optimized through cultivar selection rather than relying exclusively on fertilizer inputs [45].

### 4.5. Forage Biofortification Response Patterns and Plant Physiological Considerations

The plant-level response to foliar Zn–Fe application showed evidence of cultivar-dependent mineral accumulation. Stronger accumulation patterns for Fe than Zn are consistent with established differences in mobility and translocation behavior across micronutrients, including known limitations in Zn movement within plant tissues [4]. The observed variation among cultivars and across harvest cuts also supports the role of genotype and plant maturity in regulating micronutrient sequestration and partitioning. Similar genotype-dependent uptake patterns have been reported in other crops and legumes under agronomic enrichment strategies [16], reinforcing that biofortification outcomes are not solely a function of fertilizer dose but also of plant physiology and developmental stage.

### 4.6. Multivariate Meat-Quality Patterns and Interpretation

Multivariate analyses suggested that the enriched diet was associated with more homogeneous meat-quality profiles, including tighter clustering of individuals within treatment space. Biologically, this pattern is plausible because Zn and Fe are involved in antioxidant defense systems, enzyme function, and hemoprotein stability, all of which can influence muscle color parameters and protein matrix characteristics [46]. However, these multivariate signatures should be interpreted as supportive rather than definitive, particularly given the limited replication and the exploratory nature of the multivariate modeling. In the context of reviewer concerns, the primary emphasis should remain on core outcomes, with multivariate findings used to complement rather than replace the main performance and mineral results.

### 4.7. Implications for Smallholder Production Systems

Collectively, the results indicate that Zn–Fe biofortified alfalfa has practical potential to improve feed efficiency without compromising growth performance or meat quality in guinea pigs. The cultivar-dependent patterns observed suggest that selecting responsive alfalfa genotypes may enhance the effectiveness of agronomic biofortification strategies. Although tissue mineral deposition was not markedly improved, the combination of efficiency gains and evidence of meat-quality stabilization supports the relevance of biofortification as a feasible intervention for resource-limited guinea pig production systems where mineral supplementation options may be constrained.

### 4.8. Limitations and Research Priorities

This study has several limitations that constrain inference. First, the small sample size per factorial cell (*n* = 3) reduces statistical power and increases the probability of Type II error; therefore, non-significant effects should not be interpreted as evidence of no biological response, and even significant effects may be unstable at low replication. Second, the absence of independent replication at the treatment group level raises the possibility of pseudo-replication for group-fed variables and limits robust estimation of treatment-level effects. Third, the absence of baseline soil micronutrient data limits the interpretation of how pre-existing soil conditions may have influenced the magnitude of forage enrichment.

Future studies should incorporate (i) fully replicated factorial designs with larger sample sizes, (ii) baseline soil testing to contextualize agronomic response, and (iii) evaluation of dose optimization and alternative mineral forms to determine whether tissue mineral deposition can be enhanced while maintaining animal safety and performance.

## 5. Conclusions

This experiment showed that agronomic bio-enrichment of alfalfa with zinc and iron has the capability of enhancing the feed efficiency of guinea pigs without negatively impacting the growth and meat properties. Even though biofortification showed no significant increase in live weight gain and no change in tissue concentrations of Zn and Fe, animals fed on the enriched forage had continually better feed conversion ratios, and this suggests improved nutrient metabolism. The specific effects of plant genotypes, which are especially noticeable in Cuf 101 and California 55, underscore the significance of plant genotypes in the efficiency of mineral enrichment strategies. Multivariate analyses also indicated that the enriched diet produced more consistent meat-quality profiles and lower variability of animals, indicating greater physiological stability. All in all, the Zn–Fe biofortified alfalfa is a valid approach to enhancing production effectiveness in small-holder guinea pig systems, and there is the possibility of an outcome in food security in the rural areas. Future studies must examine increased levels of minerals, alternative sources of the nutrients Zn and Fe, and the possibility of improved methods of biofortifying to maximize the level of nutrient deposition and the nutritional value.

## Figures and Tables

**Table 1:** Summary of two-Way ANOVA results, including effect sizes.

Response Variable	Effect	df (Effect, Error)	F-Value	*p*-Value	η^2^p
Body weight	Diet	1, 32	24.60	<0.001 ***	0.435
	Time	7, 32	74.90	<0.001 ***	0.942
	Diet × Time	7, 32	1.58	0.17	0.257
Feed intake	Diet	1, 32	6.82	0.014 *	0.176
	Time	7, 32	2.65	0.028 *	0.367
	Diet × Time	7, 32	2.02	0.049 *	0.306
Feed conversion ratio	Diet	1, 32	19.10	<0.001 ***	0.374
	Time	7, 32	6.77	<0.001 ***	0.597
	Diet × Time	7, 32	0.61	0.74	0.118
Average daily gain	Diet	1, 20	7.58	0.016 *	0.275
	Time	4, 20	1.75	0.19	0.259
	Diet × Time	4, 20	2.61	0.048 *	0.343

Note: Data were analyzed using a two-way analysis of variance (ANOVA) with diet (D1 and D2) and time (measurement days) as fixed factors. For each response variable, the table reports the F statistic, associated *p* value, and partial eta squared (η^2^p) as a measure of effect size, representing the proportion of variance explained by each effect after controlling for other factors in the model. When a significant Diet × Time interaction was detected, Tukey’s honestly significant difference (HSD) post hoc test was applied to compare diets at individual time points. Statistical significance was set at *p* < 0.05. Significance levels are indicated as: * *p* < 0.05, and *** *p* < 0.001.

## Data Availability

The data presented in this study are included in the article and its Supplementary Materials. Additional datasets generated and/or analyzed during the current study are available from the corresponding author upon reasonable request.

## Supplementary Materials

The following supporting information can be downloaded at: https://www.mdpi.com/article/10.3390/ani16030392/s1, Figure S1. Multidimensional characterization of ingredient composition across starter and growth diets used in the Zn-Fe biofortification feeding trial.
