# Supplementary material for: Impact of Fe-Zn Biofortified Alfalfa on Growth Performance, Feed Efficiency, and Mineral Deposition in Guinea Pigs (Cavia porcellus) Under Smallholder Production Systems
Source: Animals (Basel). 2026 Jan 27;16(3):392. doi: 10.3390/ani16030392 (PMC12897176; doi:10.3390/ani16030392)
Supplement: Supplementary file 1 [file animals-16-00392-s001.zip › animals-4037396-supplementary.pdf]

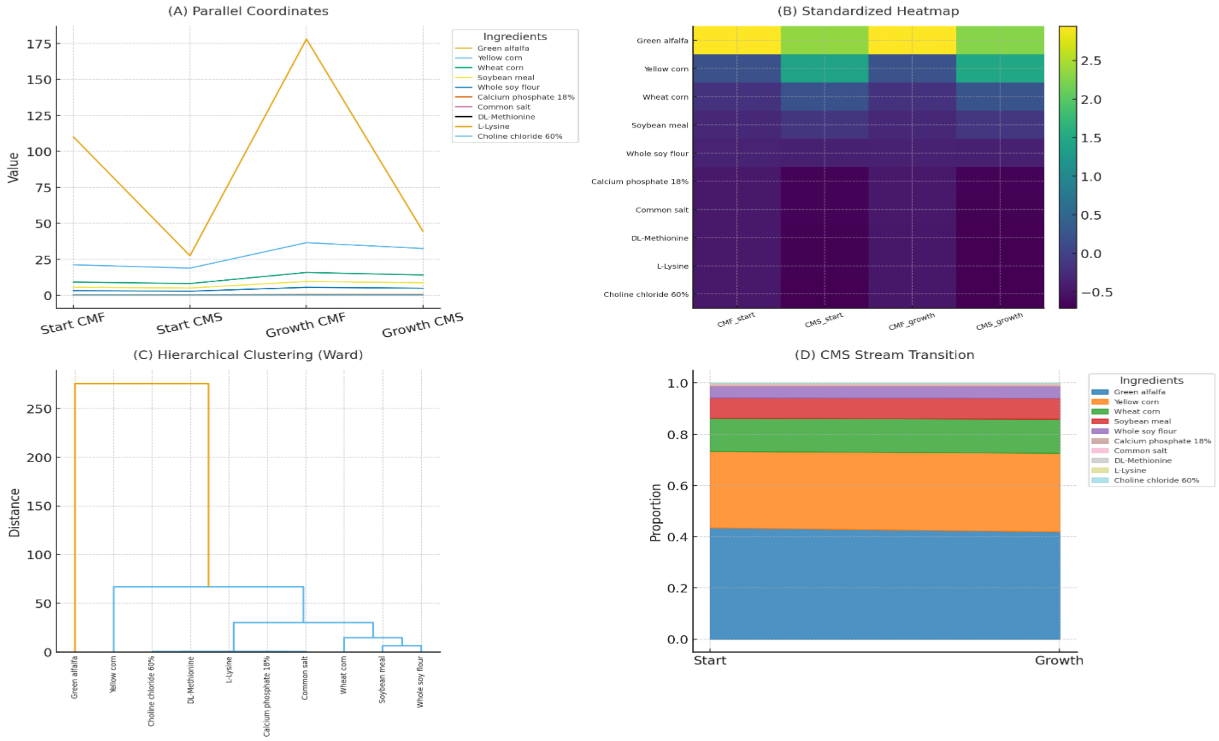

Figure S1. Multidimensional characterization of ingredient composition across starter and growth diets used in the Zn-Fe biofortification feeding trial.
